# Persistence of SARS-CoV-2 neutralizing antibodies and anti-Omicron IgG induced by BNT162b2 mRNA vaccine in patients with autoimmune inflammatory rheumatic disease: an explanatory study in Japan

**DOI:** 10.1016/j.lanwpc.2022.100661

**Published:** 2022-12-20

**Authors:** Yuta Yamaguchi, Shinichiro Nameki, Yasuhiro Kato, Ryotaro Saita, Tomoharu Sato, Sayaka Nagao, Teruaki Murakami, Yuko Yoshimine, Saori Amiya, Takayoshi Morita, Yasutaka Okita, Takahiro Kawasaki, Jun Fujimoto, Yasutaka Ueda, Yuichi Maeda, Akane Watanabe, Hyota Takamatsu, Sumiyuki Nishida, Yoshihito Shima, Masashi Narazaki, Atsushi Kumanogoh

**Affiliations:** a Department of Respiratory Medicine and Clinical Immunology, Graduate School of Medicine, Osaka University, Suita, Japan; b Department of Immunopathology, World Premier International Research Center Initiative (WPI), Immunology Frontier Research Center (IFReC), Osaka University, Suita, Japan; c Department of Medical Innovation, Osaka University Hospital, Suita, Japan; d Department of Biostatistics and Data Science, Graduate School of Medicine, Osaka University, Suita, Japan; e Center for Infectious Diseases for Education and Research (CiDER), Osaka University, Suita, Japan; f Department of Hematology and Oncology, Graduate School of Medicine, Osaka University, Suita, Japan; g Integrated Frontier Research for Medical Science Division, Institute for Open and Transdisciplinary Research Initiatives (OTRI), Osaka University, Suita, Japan; h Laboratory of Thermo-Therapeutics for Vascular Dysfunction, Graduate School of Medicine, Osaka University, Suita, Japan; i Department of Advanced Clinical and Translational Immunology, Graduate School of Medicine, Osaka University, Suita, Japan; j Japan Agency for Medical Research and Development—Core Research for Evolutional Science and Technology (AMED–CREST), Osaka University, Osaka, Japan; k Center for Advanced Modalities and DDS (CAMaD), Osaka University, Osaka, Japan

**Keywords:** SARS-CoV-2, BNT162b2 mRNA vaccine, SARS-CoV-2 Omicron variant, Humoral response, Autoimmune inflammatory rheumatic disease

## Abstract

**Background:**

Autoimmune inflammatory rheumatic disease (AIRD) patients are at high risk of the coronavirus disease 2019 (COVID-19), but the medium-term effects of immunosuppressants on vaccine efficacy are unknown. We investigated the duration of humoral responses against severe acute respiratory syndrome coronavirus 2 (SARS-CoV-2) wild-type and Omicron variant in AIRD patients administered with two doses of the BNT162b2 (Pfizer–BioNTech) vaccine.

**Methods:**

Serum-neutralizing antibody (NAb) and anti-receptor-binding domain (RBD)/spike antibody levels were measured. Short- and medium-term effects of immunosuppressants were analyzed pre-vaccination (Term 1) and 14–42 days (Term 2) and 100–200 days (Term 3) after the second vaccination.

**Findings:**

From Feb 1, 2021, to Feb 28, 2022, 439 AIRD patients and 146 healthy controls were investigated. The seropositivity rate and log_10_-NAb titers were significantly lower in AIRD patients than in controls at Terms 2 and 3. In rheumatoid arthritis patients, tumor necrosis factor-α inhibitors (TNFis) at Term 3, and older age, glucocorticoids, and abatacept at Terms 2 and 3 were risk factors for reduced responses. Anti-Omicron RBD/spike IgG levels strongly correlated with NAb titers.

**Interpretation:**

Glucocorticoids, TNFis, and abatacept treatments negatively affect the longevity of humoral responses to SARS-CoV-2, including Omicron, after two vaccine doses. These findings may inform the timing of additional vaccination for AIRD patients.

**Funding:**

Cloud Funding of Peace Winds Japan; 10.13039/501100009033Center of Innovation Program from the 10.13039/501100001700Ministry of Education, Culture, Sports, Science and Technology of Japan; 10.13039/501100001691Japan Society for the Promotion of Science KAKENHI; 10.13039/100009619Japan Agency for Medical Research and Development; 10.13039/100020408Kansai Economic Federation; Mitsubishi Zaidan; and Research Grant from 10.13039/100009619Japan Agency for Medical Research and Development—10.13039/501100003382Core Research for Evolutional Science and Technology.

**Research in context**

**Evidence before this study**

We screened studies published between Jan 1, 2020, and Aug 1, 2022, in PubMed using the terms “COVID-19”, “SARS-CoV-2”, AND “BNT162b2” AND “rheumatoid arthritis”, “systemic lupus erythematosus”, “ANCA-associated vasculitis” to find original articles and systematic reviews published in English, examining the humoral immune response against SARS-CoV-2, induced by the BNT162b2 mRNA vaccine, in patients with an autoimmune inflammatory rheumatic disease (AIRD) induced by the two doses of BNT162b2 mRNA vaccine. Most studies focused on short-term humoral immune responses within 4 months after the second BNT162b2 vaccination. These short-term observations revealed that the use of several immunosuppressants such as rituximab, mycophenolate mofetil, glucocorticoids, abatacept, and methotrexate, correlated with a decrease in SARS-CoV-2 antigen-specific antibodies. Regarding the immune response to variants of concern induced by BNT162b2, the level of antibody produced against the Delta variant was reported to be lower than that against the wild-type or Alpha variant in patients with AIRDs. However, no studies have evaluated the immune responses against the Omicron variant induced by BNT162b2 in patients with AIRDs.

**Added value of this study**

We enrolled 598 patients with AIRDs and 148 healthy controls in this study. We found that medium-term humoral immunity was attenuated in anti-neutrophil cytoplasmic antibody-associated vasculitis and rheumatoid arthritis (RA) patients treated with glucocorticoids, tumor necrosis factor-α inhibitors (TNFis), or abatacept. In patients with RA or systemic lupus erythematosus, the levels of anti-Omicron receptor-binding domain/spike IgG induced by two doses of BNT162b2 strongly correlated with neutralizing antibody titers but were lower than those of anti-SARS-CoV-2 wild-type antibodies.

**Implications of all the available evidence**

Previously, TNFis were thought to have few short-term effects on antibody titer production after vaccination, but our report reveals potential negative medium-term effects. Therefore, our results suggest that the recommended interval of consecutive vaccination for AIRD patients may differ depending on the disease and treatment. In particular, an early booster vaccination may be recommended for anti-neutrophil cytoplasmic antibody-associated vasculitis and RA patients receiving treatment with glucocorticoids, TNFis, or abatacept. Furthermore, not only were antibody titers against variants of concern lower in immunosuppressant users after two vaccinations lower, but cellular immune responses were also reduced compared to those in healthy controls, representing one of the reasons why multiple vaccinations are recommended for patients receiving immunosuppressants.

## Introduction

Preventing the spread of coronavirus disease 2019 (COVID-19) is of paramount importance. BNT162b2 (Pfizer–BioNTech), a messenger RNA (mRNA)-based vaccine, demonstrates a high efficacy rate with an acceptable safety profile.^1^ However, the efficacy of this vaccine wanes over time,^2^ and severe acute respiratory syndrome coronavirus 2 (SARS-CoV-2) variants of concern (VOCs), most recently the Omicron strain, have a negative impact on vaccine strategies.^3^

Patients with an autoimmune inflammatory rheumatic disease (AIRD), particularly those undergoing treatment with glucocorticoids (GCs), rituximab, or Janus kinase inhibitors (JAKis), are at a high risk of severe SARS-CoV-2 infection.^4^^,^^5^ To combat COVID-19, vaccination is recommended for immunocompromised patients as well as healthy individuals, and indeed, mRNA vaccines that utilize prime and booster doses are associated with a lower rate of SARS-CoV-2 infection and severity in cases of vaccinated AIRD patients than in unvaccinated cases.^6^^,^^7^ Recent studies have shown that several immunosuppressants (e.g., rituximab, methotrexate [MTX], mycophenolate mofetil [MMF], abatacept [ABT], and GCs) are associated with an impaired humoral response despite booster immunization.^8^, ^9^, ^10^ However, these data are from a relatively short observation period, and the medium- to long-term effects of immunosuppressive treatments for vaccine effects have rarely been reported. Furthermore, the duration of immune responses against VOCs, including the Omicron variant, remains unclear.

In the face of challenges, such as the decline in humoral immune response over time and the emergence of new variants (e.g., Omicron), a third or fourth vaccination, which can effectively increase neutralizing antibody (NAb) titers, was approved.^11^^,^^12^ However, there is no consensus on the priority and dosing intervals for additional vaccination, considering that vaccine efficacy may vary among patients or treatments. Understanding the medium- to long-term impacts of immunosuppressive treatments on BNT162b2 mRNA vaccine efficacy could be helpful for physicians to clarify which patients should be prioritized for additional vaccination.

The aim of this study was to evaluate the medium-term influence of individual immunosuppressive treatments by longitudinally evaluating SARS-CoV-2 neutralizing and anti-Omicron antibody levels after the administration of two doses of BNT162b2 in patients with AIRDs.

## Methods

### Study design and participants

This study was a prospective longitudinal study from Feb 1, 2021, to Feb 28, 2022. Participants included patients diagnosed with AIRDs who visited the Department of Clinical Immunology at Osaka University Hospital participated in this study. Written informed consent was obtained from all patients, and the study was approved by the local ethics committee of Osaka University Hospital (IRB no. 20118). The vaccines were administered as part of a clinical practice, not a research study, and the vaccination schedule and intervals were dependent on patient discretion, with the attending physician judging whether or not to vaccinate with BNT162b2. The vaccine dose (30 μg/dose) and site of administration (deltoid muscle) were chosen according to the manufacturer's instructions. Data on the diagnosis, treatment, demographics (age, sex, body mass index, smoking status, medical history, comorbidities, and history of SARS-CoV-2 infection), and vaccination date of patients were extracted from the electronic medical records of Osaka University Hospital.

All healthy controls (HCs) were healthcare workers recruited via convenience sampling and voluntarily participated in this study. Written informed consent was obtained from all HCs. HCs also received two doses of the BNT162b2 mRNA vaccine in the deltoid muscle, more than 3 weeks apart (30 μg/dose).

Individuals meeting any of the following criteria were excluded: (1) those who withdrew consent, (2) those with no vaccination date on their electronic medical record, (3) those with a history of SARS-CoV-2 infection, and (4) those whose pre-vaccination antibody titer was above the cut-off value. Only participants who received two doses of the vaccine were included in the analysis. If a participant was infected with SARS-CoV-2 or received a third dose of vaccine during the observation period, subsequent data were excluded from the analysis.

### Samples

AIRD patient sera were obtained without additional blood sampling by collecting all residual specimens after outpatient clinical testing to assess humoral immunity before the first vaccination (−294 to −21 days), before (−30 to 0 days) the second vaccination, and after the second vaccination (1–13, 14–42, 43–99, 100–200, and 201–301 days). Peripheral blood mononuclear cells (PBMCs) were obtained from patients who consented to have additional blood sampling. Control serum and PBMC samples were collected from healthy healthcare workers during the same period depending on their availability. Serum samples and PBMCs isolated from whole blood samples by density gradient centrifugation were stored at −80 °C and in liquid nitrogen, respectively, until use.

### Serum SARS-CoV-2 NAb quantification

Serum SARS-CoV-2 NAb concentrations were measured using an iFlash3000 (YHLO Biotech Co., Ltd., Shenzhen, China, YH-C6111) fully automated chemiluminescent immunoassay analyzer and an iFlash-2019-nCoV NAb kit (YHLO Biotech Co., Ltd., YH-C86109), following the manufacturer's protocols. The kit is a one-step competitive assay that measures the binding inhibitory activity of antibodies to the SARS-CoV-2 receptor-binding domain (RBD) that binds to angiotensin-converting enzyme 2, the receptor for viral entry. A calibration curve was created using the four-point calibrator supplied in the kit. The high-throughput results were provided as inhibition activity in arbitrary units/mL (AU/mL), and considered seropositive if the NAb titer was above 10 AU/mL.

### Serum SARS-CoV-2 antigen-specific antibody quantification

The serum concentrations of SARS-CoV-2 antigen-specific antibodies, including those recognizing mutant variants, were measured using a V-PLEX SARS-CoV-2 Panel 22 (IgG) Kit and a Panel 24 (IgG) Kit (Meso Scale Discovery Inc., Rockville, MD, USA; K15559U and K15575U) following the manufacturer's protocols. In brief, 150 μL Blocker A solution was added to each well of a 96-well plate and incubated at room temperature (18–25 °C) for 30 min with shaking (700 rpm). After washing three times, 50 μL of diluted serum (1000-fold), calibrators, and controls were added to each well. After 2 h of incubation at room temperature with shaking (700 rpm), the plate was washed three times. Detection Antibody Solution was applied to the plate and incubated at room temperature for 1 h with shaking (700 rpm). After washing three times, the plate was filled with 150 μL of Read Buffer and analyzed using a MESO QuickPlex SQ 120 (Meso Scale Discovery Inc., AI0AA-0). The Panel 22 Kit is a multiplex serology assay for IgG antibodies to the SARS-CoV-2 RBD (wild-type, Alpha, Beta, Gamma, Delta, and Omicron). The Panel 24 Kit is a multiplex serology assay for IgG antibodies to SARS-CoV-2 Spike (wild-type, Alpha, Beta, Gamma, Delta, and Omicron) and Nucleocapsid (wild-type). The cut-off values of IgG antibodies against the SARS-CoV-2 RBD (wild-type), Spike (wild-type), and Nucleocapsid (wild-type) were 538, 1960, and 5000 AU/mL, respectively. The cut-off values for IgG antibodies against mutant variants were not defined.

### T-cell response using enzyme-linked immunospot (ELISPOT) assay

SARS-CoV-2 antigen-specific T-cell responses were assessed using a Human Interferon (IFN)-γ Single-Color ELISPOT (Cellular Technology Limited [CTL], Shaker Heights, OH, USA; hIFNg-1M), according to the manufacturer's instructions. Briefly, 100,000 PBMCs/well were incubated with 0.5 μg/mL SARS-CoV-2 Spike PepMix (JPT, PM-WCPV-S-1-2 [wild-type], PM-SARS2-SMUT08-1 [Omicron variant]) for 19 h at 37 °C under 5% CO_2_. Dynabeads Human T-Activator CD3/28 (Gibco, Waltham, MA, USA, 11131D) was used for positive control wells. Images of the plates were captured using an ImmunoSpot S6 MACRO Analyzer (CTL) and ImmunoCapture software (version 7.0.7.3). The number of spot-forming cells (SFCs) was automatically calculated using ImmunoSpot (version 7.0.28.5). The number of spots in the negative control wells (containing only dimethyl sulfoxide) was subtracted from the number of spots in the test wells; the results are displayed as SFCs.

### Covariates

Explanatory variables were as follows: sex, age, disease category (rheumatoid arthritis [RA]; systemic lupus erythematosus [SLE]; anti-neutrophil cytoplasmic antibody [ANCA]-associated vasculitis [AAV]; large vessel vasculitis [LVV]; Sjögren syndrome [SjS]; systemic sclerosis [SSc]; mixed connective tissue disease [MCTD]; IgG4-related disease [IgG4RD]; polymyalgia rheumatica [PMR]; and Behçet disease [BD]), and immunosuppressant category (GCs; MTX; cyclosporine A [CyA]; tacrolimus [TAC]; MMF; azathioprine [AZA]; tumor necrosis factor-α inhibitors [TNFis; infliximab, etanercept, adalimumab, certolizumab pegol, and golimumab]; interleukin [IL]-6 receptor inhibitors [IL-6Ris; tocilizumab, sarilumab]; ABT; Janus kinase inhibitors [JAKis; tofacitinib, baricitinib, upadacitinib, and peficitinib]; and belimumab [BEL]).

### Statistical analysis

Descriptive statistics are reported as the median and interquartile range (IQR; with full range for age) for continuous variables, frequency, or proportion for categorical variables. Mann–Whitney U and Fisher's exact tests were used to analyze differences in demographic background, NAb titer, and seropositivity between patient and control groups. Non-parametric analyses of antigen-specific IgG titers or T-cell responses were performed using Friedman and Dunn's multiple comparisons tests, Kruskal–Wallis and Dunn's multiple comparison tests, or Wilcoxon matched-pairs signed rank test. Correlation analyses were performed using Spearman's tests. Disease distribution and the relationship with medication are represented in contingency tables. Multiple regression analyses with a single model were performed for the patient and control groups together to assess the relationships between NAb and explanatory variables as noted above for each period (Term 1, before the first vaccination; Term 2, 14–42 days after the second vaccination; and Term 3, 100–200 days after the second vaccination). The categories with five or more individuals in all three terms (Terms 1–3) were included in the regression analyses. In all regression models, the common log-transformed NAb titers were used as independent variables. The adjusted mean NAb titer for each disease group or treatment category of patients with AIRDs was calculated with the “emmeans” package (version 1.7.4–1) in R using estimates of the models. The estimated mean values and 95% confidence intervals (CIs) were compared to those of HCs. Missing data were handled using a complete-case analysis. Statistical significance was set at p < 0.05, and multiplicity was not adjusted for because this study was exploratory. The graphical representations and statistical analyses were executed using GraphPad Prism (version 9.3.1; GraphPad Software Inc., San Diego, CA, USA), JMP Pro (version 16.0.0; SAS Institute Inc., Cary, NC, USA), or R (version 4.0.2; R Development Core Team, Vienna, Austria) within RStudio (RStudio Corp., Boston, MA, USA). Illustrations were created by BioRender.com.

### Role of the funding sources

The funders of this study were not involved in the study design; the collection, analysis, and interpretation of data; the writing of the report; or the decision to submit the paper for publication.

## Results

### Study sample and baseline characteristics

**Fig. 1:**
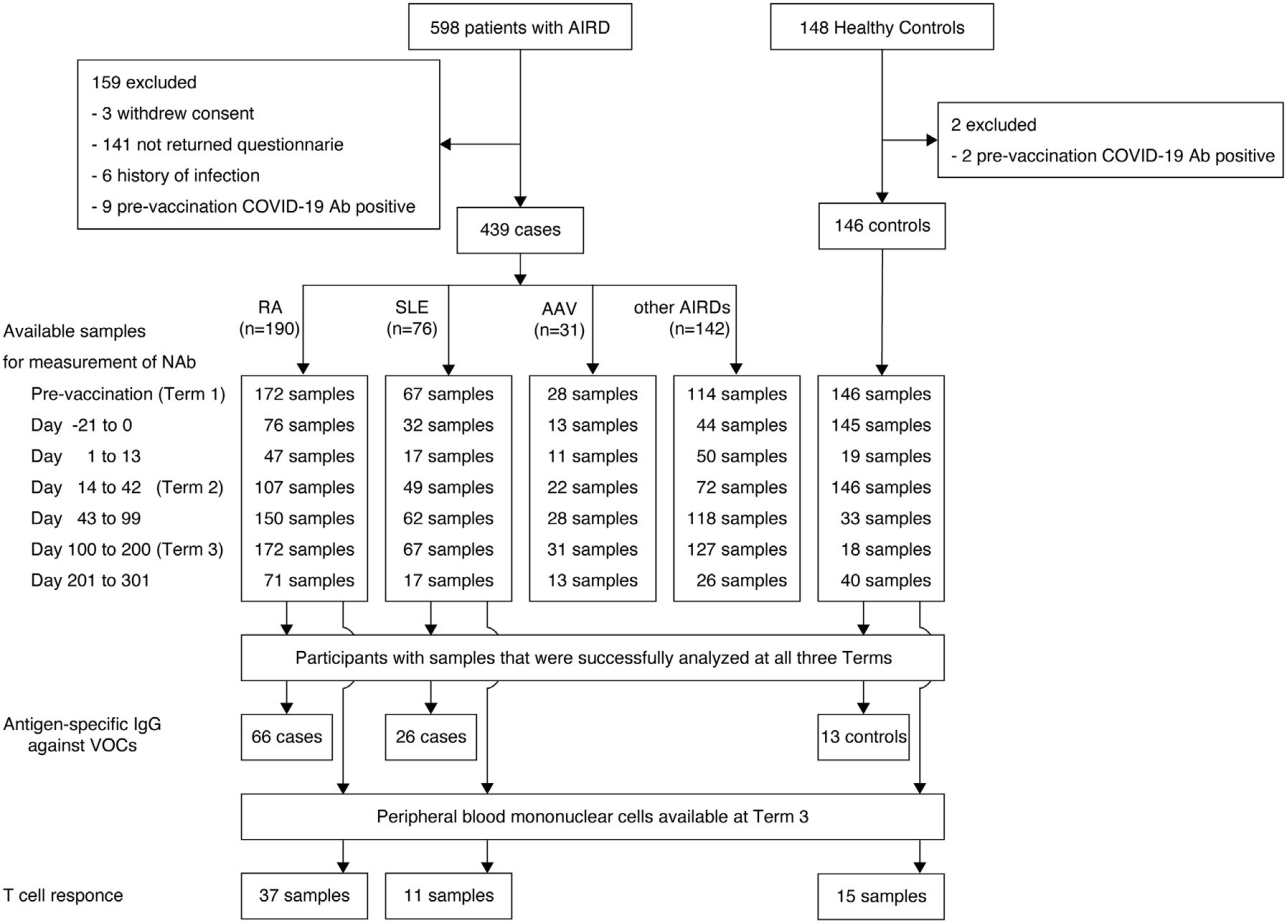
Study population for the analysis of the BNT162b2 vaccination. AAV, anti-neutrophil cytoplasmic antibody-associated vasculitis; Ab, antibody; AIRD, autoimmune inflammatory rheumatic disease; COVID-19 Ab, SARS-CoV-2 neutralizing Ab or anti-SARS-CoV-2 Nucleocapsid IgG; NAb, SARS-CoV-2 neutralizing Ab; RA, rheumatoid arthritis; SLE, systemic lupus erythematosus; VOCs, variant of concerns.

A total of 598 patients with AIRDs and 148 HCs were enrolled in this study. After exclusion according to the stated criteria, 439 patients and 146 HCs were finally included in the analysis (Fig. 1). Three patients and two HCs were infected with SARS-CoV-2, and 54 patients and 28 HCs received a third dose of vaccine during the observation period. Their samples were excluded from the subsequent analysis. The diagnoses and treatment of the included patients are listed in Table 1, and the distribution of diagnoses and medications are shown in Supplemental Table S1. Samples were collected from patients and HCs on their availability, so sample numbers varied by period or disease (Supplemental Fig. S1).

**Table 1:** Characteristics of patients with AIRDs and healthy controls.

	Patients (n = 439)	Healthy controls (n = 146)	p value
Demographic characteristics			
Age (years), median (IQR [range])	65 (52–74 [20–93])	38 (33–47 [21–65])	<0.0001
Male	93 (21.2)	81 (55.5)	<0.0001
BMI (kg/m^2^), median (IQR)	21.7 (19.2–24.3)	22.2 (20.1–24.7)	0.2303
Smoking status			
Never	304 (69.2)	123 (84.1)	0.0013
Former	108 (24.6)	20 (13.7)	
Current	27 (6.2)	3 (2.1)	
Comorbidities			
Obesity (BMI >25 kg/m^2^)	89 (20.3)	31 (21.2)	0.814
Hypertension	72 (16.4)	8 (5.5)	0.0005
Dyslipidemia	36 (8.2)	10 (6.8)	0.723
Diabetes	17 (3.9)	0 (0.0)	0.0097
Hyperuricosuria	10 (2.3)	3 (2.1)	>0.999
Cardiac disease	63 (14.4)	5 (3.4)	0.0002
Stroke	30 (6.8)	0 (0.0)	0.0003
Malignancy	52 (11.8)	0 (0.0)	<0.0001
Lung disease	120 (27.3)	9 (6.2)	<0.0001
AIRDs			
Rheumatoid arthritis	190 (43.3)	··	
Systemic lupus erythematosus	76 (17.3)	··	
ANCA-associated vasculitis	31 (7.1)	··	
Large vessel vasculitis	14 (3.2)	··	
Sjögren syndrome	25 (5.7)	··	
Systemic sclerosis	19 (5.0)	··	
Mixed connective tissue disease	15 (3.4)	··	
IgG4 related disease	12 (2.7)	··	
Polymyalgia rheumatica	11 (2.5)	··	
Behçet disease	11 (2.5)	··	
Others^a^	35 (8.0)	··	
Treatment for AIRDs			
Glucocorticoid	230 (52.4)	··	
dose (mg/d), median (IQR)	5.0 (3–6.59)	··	
Methotrexate	152 (34.6)	··	
dose (mg/week), median (IQR)	8.0 (6–10)	··	
Cyclosporine	10 (2.3)	··	
dose (mg/d), median (IQR)	100 (62.5–140)	··	
Tacrolimus	36 (8.2)	··	
dose (mg/d), median (IQR)	2 (1–3)	··	
Mycophenolate mofetil	24 (5.5)	··	
dose (mg/d), median (IQR)	1500 (562.5–2000)	··	
Azathioprine	32 (7.3)	··	
dose (mg/d), median (IQR)	75 (50–100)	··	
TNF-α inhibitors	42 (9.6)	··	
IL-6R inhibitors	58 (13.2)	··	
Abatacept	19 (4.33)	··	
JAK inhibitors	13 (3.0)	··	
Belimumab	14 (3.2)	··	
No therapy	55 (12.5)	··	

n (%) presented unless otherwise specified.

ANCA, anti-neutrophil cytoplasmic antibody; AIRD, autoimmune inflammatory rheumatic disease; BMI, body mass index; IL-6R, interleukin-6 receptor; IQR, interquartile range; JAK, Janus kinase; TNF, tumor necrosis factor.

a Adult-onset Still's disease (n = 4), anti-phospholipid antibody syndrome (n = 1), ankylosing spondylitis (n = 4), common variable immunodeficiency (n = 4), polyarteritis nodosa (n = 2), psoriatic arthritis (n = 5), relapsing polychlorides (n = 2), RS3PE syndrome (n = 2), SAPHO syndrome (n = 2), Castleman disease (n = 2), inflammatory myositis (n = 6), and necrotizing immune-mediated myopathy (n = 1).

### Longitudinal observation of SARS-CoV-2 NAb titers

**Fig. 2:**
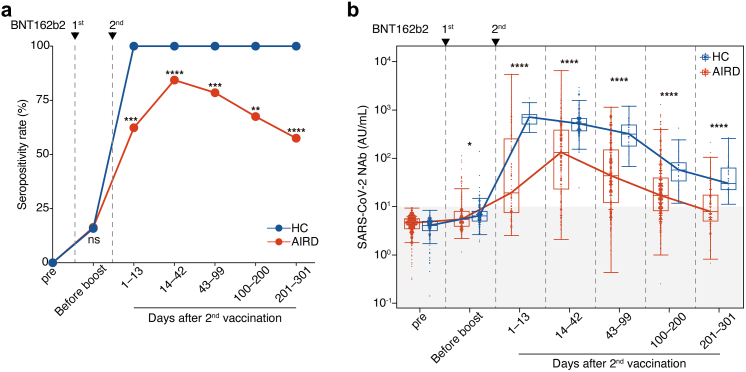
Longitudinal observation of NAb titers against SARS-CoV-2 in patients with AIRDs after BNT162b2 vaccination. (a) The seropositivity rate of SARS-CoV-2 NAb in HCs (blue) and patients with AIRDs (red) over time after the first and second vaccinations. Dotted lines and arrows indicate the timing of the first and second vaccinations. Serum samples were considered seropositive when the NAb titers were higher than 10 AU/mL. Fisher's exact test was used to assess significant differences in the seropositivity rate between HCs and patients with AIRDs at each period. ∗p < 0.05, ∗∗p < 0.01, ∗∗∗p < 0.001, ∗∗∗∗p < 0.0001. (b) Serum SARS-CoV-2 NAb titers induced by the BNT162b2 vaccine over time in HCs (blue) and patients with AIRDs (red). The Y-axis represents a log scale. The gray shaded area represents NAb titers below the cut-off for positivity (<10 AU/mL). Arrows indicate the timing of the first and second vaccinations. The boxplots show medians (middle line) and the first and third quartiles, whereas the whiskers indicate 95% CIs. The Mann–Whitney test was performed to evaluate significant differences between patients with AIRDs and HCs at each period. ∗p < 0.05, ∗∗p < 0.01, ∗∗∗p < 0.001, ∗∗∗∗p < 0.0001. AIRD, inflammatory autoimmune rheumatic disease; HC, healthy control; NAb, neutralizing antibody.

Although the seropositivity rate in HCs was consistently 100% in each period after the second vaccination, the rate in patients with AIRDs was consistently lower than that in HCs after the second vaccination (Fig. 2a). As in HCs, NAb titers in patients with AIRDs decreased gradually, but patients with AIRDs showed higher IQR values in each period than HCs (Fig. 2b), suggesting the presence of factors influencing the high diversity NAb titers in patients with AIRDs. However, age, sex, disease category, and treatment differed widely among participants (Table 2; Supplemental Tables S1 and S2).

**Table 2:** Demographic characteristics of patients with AIRDs and healthy controls.

	Age, median (IQR [range])	Male, n (%)	BMI (kg/m^2^), median (IQR)
Healthy controls (n = 146)	38 (33–47 [21–65])	81 (55.5)	22.2 (20.1–24.7)
All patients with AIRDs (n = 439)	65 (52–74 [20–93])	93 (21.2)	21.7 (19.2–24.3)
Rheumatoid arthritis	69 (58–76.3 [32–85])	38 (20.0)	21.2 (19.1–24.1)
Systemic lupus erythematosus	49.5 (41.3–60 [20–84])	9 (11.8)	21.1 (19.3–23.6)
ANCA-associated vasculitis	70 (60–77 [35–84])	13 (41.9)	22.2 (19.8–24.8)
Large vessel vasculitis	57 (46.5–75.5 [24–90])	2 (14.3)	19.7 (18.4–25.2)
Sjögren syndrome	67 (54–72 [35–78])	0 (0.0)	24.8 (20.7–27.4)
Systemic sclerosis	67 (61–76 [45–79])	3 (15.8)	20.0 (18.7–23.4)
Mixed connective tissue disease	55 (42–72 [31–76])	0 (0.0)	23.0 (18.1–24.4)
IgG4 related disease	70.5 (59.5–74 [42–76])	7 (58.3)	21.8 (19.9–23.8)
Polymyalgia rheumatica	77 (70–83 [66–93])	1 (9.1)	21.9 (19.7–23.8)
Behçet disease	50 (46–60 [44–74])	7 (63.6)	22.6 (21.8–24.3)
Others^a^	62 (49–72 [28–83])	13 (37.1)	22.1 (18.9–24.9)

ANCA, anti-neutrophil cytoplasmic antibody; AIRD, autoimmune inflammatory rheumatic disease; BMI, body mass index; IQR, interquartile range.

a Adult-onset Still's disease (n = 4), anti-phospholipid antibody syndrome (n = 1), ankylosing spondylitis (n = 4), common variable immunodeficiency (n = 4), polyarteritis nodosa (n = 2), psoriatic arthritis (n = 5), relapsing polychlorides (n = 2), RS3PE syndrome (n = 2), SAPHO syndrome (n = 2), Castleman disease (n = 2), inflammatory myositis (n = 6), and necrotizing immune-mediated myopathy (n = 1).

### Differences in NAb titers between patient and control groups

**Fig. 3:**
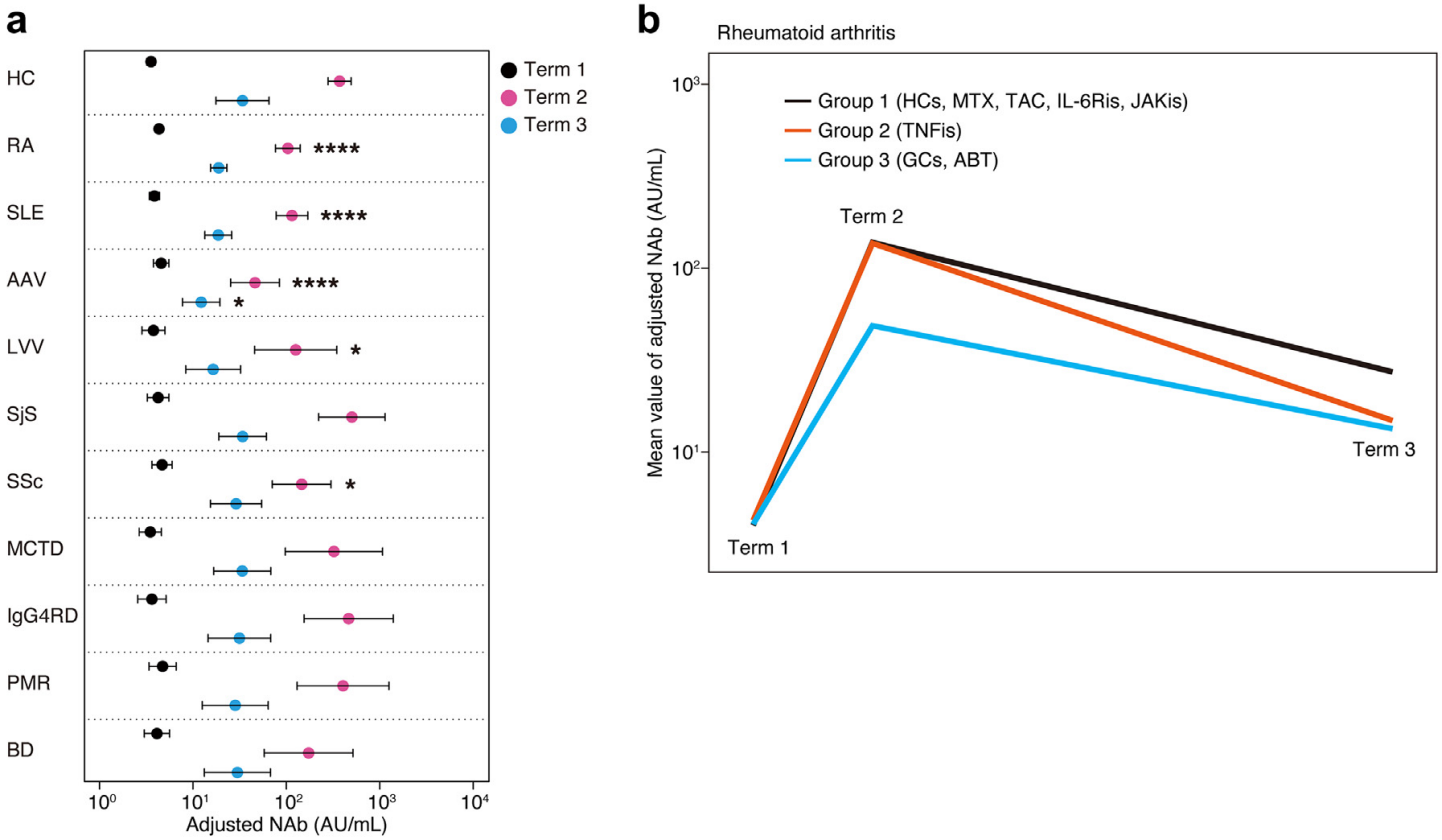
Identification of risk factors for reduced NAb titers in patients with AIRDs. (a) Adjusted mean values of SARS-CoV-2 NAb (adjusted NAb) titers induced by BNT162b2 in HCs and patients with AIRDs, taking into account age, sex, and diagnosis as covariates. The means were adjusted following multiple regression analysis. The X-axis is represented on a log scale. The error bar indicates the 95% CI. Tests for differences in adjusted mean were performed to compare diagnosis covariates with HCs. ∗p < 0.05, ∗∗p < 0.01, ∗∗∗p < 0.001, ∗∗∗∗p < 0.0001. (b) Classification by significant differences in adjusted mean NAb titers represented in Supplemental Fig. S2a: Group 1 (MTX, TAC, IL-6Ris, and JAKis): no difference compared with HCs; Group 2 (TNFis): significant difference compared with HCs only at Term 3; Group 3 (GCs and ABT): significant difference compared with HCs only at Term 2 and Term 3. Each colored line represents the mean value of adjusted NAb for each group (black, Group 1; orange, Group 2; blue, Group 3). AAV, anti-neutrophil cytoplasmic antibody-associated vasculitis; ABT, abatacept; AIRD, autoimmune inflammatory rheumatic disease; BD, Behçet disease; GCs, glucocorticoids; HC, healthy control; IgG4RD, IgG4-related disease; IL-6Ris, interleukin (IL)-6 receptor inhibitors; JAKis, Janus kinase inhibitors; LVV, large vessel vasculitis; MCTD; mixed connective tissue disease; MTX, methotrexate; NAb, neutralizing antibody; PMR, polymyalgia rheumatica; RA, rheumatoid arthritis; SjS, Sjögren syndrome; SLE, systemic lupus erythematosus; SSC, systemic sclerosis; TAC, tacrolimus; TNFis, tumor necrosis factor-α inhibitors. Term 1, pre-vaccination; Term 2, 14–42 days after the second vaccination; Term 3, 100–200 days after the second vaccination.

According to the results of the longitudinal assessment of NAb titers in patients with AIRDs, we focused on Term 1, Term 2 and Term 3 for subsequent analyses for the following reasons: Term 1 was the pre-vaccination period; Term 2 (14−42 days after the second vaccination) was the period that showed the maximum NAb titers in the AIRDs group, and Term 3 (100−200 days after the second vaccination) was the longest post-vaccination period among the periods that included enough samples for further analyses (Fig. 2b and Supplemental Fig. S1). We performed multiple regression analysis to assess the short- and medium-term effects of the two doses of vaccination on NAb titers in different patient groups, and then for each time point assessed estimated marginal means adjusting for age, sex, and each disease group. The analysis revealed that the adjusted mean values of log_10_-NAb at Term 2 were significantly lower in the category of RA, SLE, AAV, and SSc than in HCs; at Term 3, only the category of AAV showed significantly lower NAb titers than HCs (Fig. 3a and Supplemental Table S3).

### Effect of immunosuppressants on the humoral immunity induced in patients with RA, SLE, and AAV

Various types of immunosuppressants were prescribed for patients with RA, SLE, and AAV (Supplemental Tables S4–S6). We then focused on RA and SLE, which had sufficient sample sizes, and performed multiple regression analysis to examine the differences in the effect of each treatment on NAb titers in patients with RA or SLE. Regarding the effects of treatment for RA, we performed multiple regression analysis, considering age, sex, diagnosis of RA, and treatments. The analysis at Term 2 showed significantly lower adjusted mean log_10_-NAb in patients under treatment with GCs, or ABT. MTX had a similar trend with lower adjusted mean log_10_-NAb at Term 2, there was no statistical significance (p = 0.060) (Supplemental Table S7 and Supplemental Fig. S2a). The same analysis at Term 3 revealed that the adjusted mean log_10_-NAb values in patients with RA under treatment with GCs, TNFis, or ABT were significantly lower than those in HCs (Supplemental Table S7 and Supplemental Fig. S2a). The differences in the efficacy of treatment on NAb titers were categorized into three groups: Group 1 showed that some treatments had no significant effect on NAb titers at both Terms 2 and 3; Group 2 showed that TNFis reduced NAb titers at Term 3, but not at Term 2; Group 3 showed that GCs and ABT caused lower NAb titers at both Terms 2 and 3 (Fig. 3b).

We also performed the same analysis for patients with SLE using covariates of age, sex, and treatments. Although we could not include the diagnosis of SLE as a covariate due to the overlap between the diagnosis and the treatment of GCs, the analysis at Term 2 showed significantly lower adjusted mean log_10_-NAb values in not only patients undergoing treatment with GCs monotherapy but also GCs with TAC, MMF, or BEL combination therapy, in comparison with non-treated HCs (Supplemental Table S8 and Supplemental Fig. S2b).

In patients with AAV, there were not enough samples to evaluate the estimated means of NAb titers for each treatment despite the significant influence on NAb titers at both Terms 2 and 3 (Fig. 3a). Instead, we compared unadjusted NAb titers in the patients treated with GCs monotherapy and treated with GC and AZA combination therapy. Thus, there was no significant difference in log_10_-NAb values shown between patients undergoing GC monotherapy and those undergoing treatment with GCs and AZA at both Terms 2 and 3 (Supplemental Fig. S2c); patients undergoing GC monotherapy were older than those undergoing GCs and AZA combination therapy (median 64 vs. 77 years, p = 0.0184).

### Medium-term humoral immune response to the Omicron variant in patients with RA and SLE

**Fig. 4:**
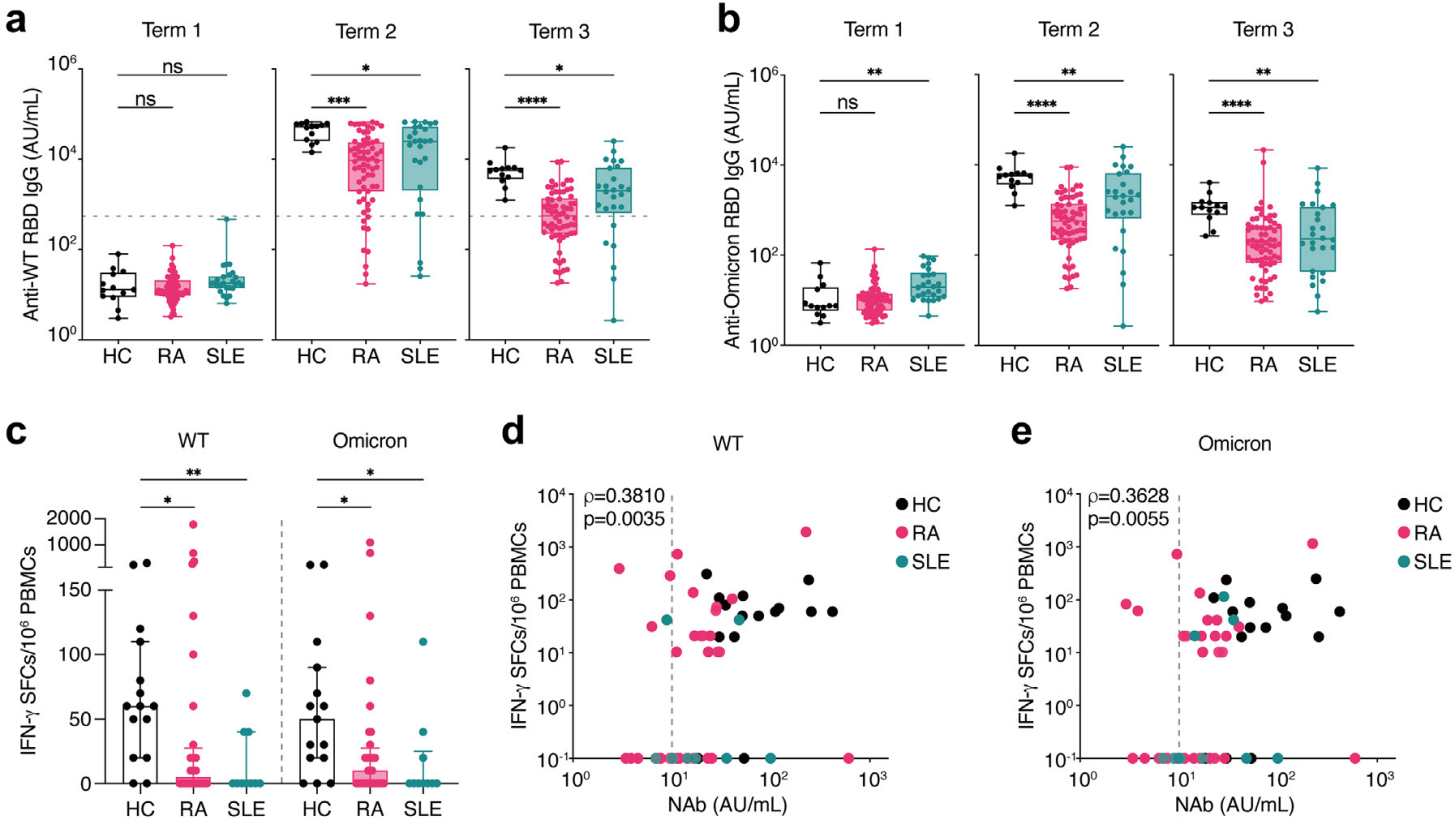
Humoral and cellular responses to the SARS-CoV-2 Omicron variant (B.1.1529) in patients with RA and SLE after two doses of BNT162b2. (a, b) Levels of anti-SARS-CoV-2 wild-type (a) and Omicron (b) RBD IgG induced by the BNT162b2 vaccine in HCs (n = 13, black), patients with RA (n = 66, red), and patients with SLE (n = 26, blue). Bars and error bars represent the median and IQR, respectively. The dotted line indicates the cut-off value for anti-WT RBD IgG positivity (538 AU/mL), according to the manufacturer's protocol. The cut-off value for anti-Omicron RBD IgG was not defined. Kruskal–Wallis and Dunn's multiple comparison tests were performed to evaluate the significant differences in antigen-specific antibody titers of patients with RA and SLE compared with those of HCs at each term. ∗p < 0.05, ∗∗p < 0.01, ∗∗∗p < 0.001, ∗∗∗∗p < 0.0001. (c) Long-term T-cell responses to SARS-CoV-2 WT (left column) and Omicron (right column) spike protein peptides in HCs (n = 15, black) and patients with RA (n = 37, red) and SLE (n = 11, blue) at Term 3. Kruskal–Wallis and Dunn's multiple comparison tests was performed to evaluate the significant differences in T-cell responses of patients with RA and SLE to WT and Omicron spike protein peptides compared with those of HCs. ∗p < 0.05, ∗∗p < 0.01, ∗∗∗p < 0.001, ∗∗∗∗p < 0.0001 (d, e) Correlation of T-cell responses to SARS-CoV-2 WT (D, left; n = 57) and Omicron (E, right; n = 57) with SARS-CoV-2 NAb titers. The X- and Y-axes are represented on a log scale. For the visualization of data on a log scale, values = 0 are represented by 0.1. The dotted lines represent the NAb positivity cut-off levels (<10 AU/mL). Spearman's test was used to evaluate the significant differences. HC, healthy control; NAb, neutralizing antibody; PBMC, peripheral blood mononuclear cell; RA, rheumatoid arthritis; RBD, receptor-binding domain; SLE, systemic lupus erythematosus; SFC, spot-forming cell; WT, SARS-CoV-2 wild-type. Term 1 (black), pre-vaccination; Term 2 (red), 14–42 days after the second vaccination; Term 3 (blue), 100–200 days after the second vaccination.

The BNT162b2 mRNA vaccine encodes the SARS-CoV-2 wild-type spike protein, but the prevalence of mutant variants is problematic due to the reduced efficacy of vaccination.^3^ Therefore, we next investigated humoral immune responses to the SARS-CoV-2 wild-type and VOCs (Alpha, B.1.17; Beta, B.1.351; Gamma, P.1; Delta, B.1.617.2; Omicron, B.1.1.529) by analyzing antigen-specific IgG (anti-RBD and anti-spike IgG) titers induced by two doses of BNT162b2 in HCs (n = 13) and patients with RA (n = 66) or SLE (n = 26; Fig. 1). The characteristics of these patients and HCs are presented in Supplemental Table S9. Comparing the differences in antigen-specific antibody titers against the wild-type and VOCs for each individual, we found that antibody titers against Omicron were significantly lower than those against the wild-type in patients with RA and SLE, as well as in HCs (Supplemental Fig. S3a). We also found that the levels of antigen-specific IgG against both wild-type and Omicron were strongly correlated with the NAb titers (Supplemental Figs. S3b and c). The levels of anti-Omicron RBD/spike IgG were significantly lower in patients with RA and SLE than in HCs (Fig. 4a and b). These results suggest that the production of NAb against Omicron was induced by BNT162b2 in patients with RA and SLE, but its activity was lower than that in HCs.

### Medium-term cellular immune response to the Omicron variant in patients with RA and SLE

Finally, we investigated the medium-term memory of T-cell responses against the SARS-CoV-2 wild-type and Omicron variant using an ELISPOT assay of PBMCs collected from HCs (n = 15) and patients with RA (n = 37) or SLE (n = 11) at Term 3. Their characteristics are listed in Supplemental Table S10. The T-cell response to the Omicron variant was comparable to that to the wild-type both in HCs and patients with RA and SLE (Supplemental Fig. S3d). However, the responses in patients with RA and SLE were significantly reduced compared with those in HCs (Fig. 4c). Overall, the NAb titers correlated with the number of spots; however, a discrepancy between NAb titers and the number of spots was observed in patients with RA and SLE (Fig. 4d and e).

## Discussion

Our study evaluated the short- and medium-term humoral immune responses induced by two doses of BNT162b2 in patients with AIRDs by analyzing serum SARS-CoV-2 NAb titers. Most studies regarding the effect of the SARS-CoV-2 vaccine on patients with AIRDs have been limited to relatively short-term observations within a few weeks or months after vaccination.^8^^,^^10^ As previously reported,^8^^,^^10^ our short-term observations showed a high seropositivity rate of 84.4% in patients with AIRDs at 14–42 days after the two doses of BNT162b2; however, our medium-term observations showed that the positivity rate decreased to 57.5% at more than 201 days after the second vaccination. BNT162b2 vaccine-induced humoral immune responses are influenced by various immunosuppressive treatments.^8^^,^^10^ In fact, 87.5% of the patients with AIRDs in this study were treated with immunosuppressants. Our multiple regression analysis indicated that immunosuppressants such as GCs, TNFis, and ABT may be negatively associated with the induction of prolonged humoral immune responses. These findings suggest that such treatments cause reduced effectiveness against SARS-CoV-2 infections.

TNF-α is one of the most vital cytokines involved in various inflammatory pathologies, and its inhibitors (TNFis) are widely used in the treatment of inflammatory diseases.^13^ Additionally, TNF-α plays an crucial role in the maturation of the humoral immune responses.^14^ Although there is concern regarding the impact of TNFis on the immunogenicity of vaccines,^15^ studies investigating the effects of TNFis on the response to influenza vaccination have yielded controversial results.^16^^,^^17^ Regarding the SARS-CoV-2 mRNA vaccine, the use of TNFis has little effect on the humoral immune response in the relatively short-term (2–6 weeks).^8^^,^^10^ However, TNFis may have a negative impact on the maintenance of humoral responses (5–6 months).^18^^,^^19^ Although these studies have included patients treated with TNFis for several different inflammatory diseases, such as RA, psoriatic arthritis and inflammatory bowel disease, more detailed analyses focusing on specific diseases have not been conducted. Nevertheless, there is only limited evidence of an association between the use of TNFis and medium- or long-term humoral immune responses to vaccines; therefore, further analysis, including that of the effects on other vaccines, is warranted.

ABT inhibits optimal T-cell activation by blocking costimulatory signals via the interaction between CD80/86 on antigen-presenting cells and CD28 on T cells.^20^ Inactivated T cells cannot increase their expression of CD40 ligand; thus, they fail to activate B cells via CD40.^21^ ABT also binds to CD80/86 expressed on B cells and inhibits antibody production,^22^^,^^23^ which means that ABT can affect humoral immune responses both directly and indirectly. These mechanisms may result in the reduced immunogenicity of vaccines such as pneumococcal,^24^ influenza,^25^ and SARS-CoV-2 mRNA vaccines^26^ for patients undergoing treatment with ABT. As previously reported, in our short- and medium-term observations, patients undergoing treatment with ABT had lower adjusted NAb titers than HCs. A third vaccine dose has been reported to be effective for some of low responders.^12^ In regard to belatacept, a second-generation form of CTLA4-Ig/ABT used for kidney transplant recipients, the efficacy of a third vaccine dose for humoral response is controversial.^27^^,^^28^ It remains unclear how effective the third vaccine is for patients undergoing treatment with ABT.

We also obtained additional data on adaptive immunity to SARS-CoV-2 variants in patients with AIRDs receiving the BNT162b2 mRNA vaccine, which encodes the SARS-CoV-2 wild-type spike protein. It is necessary for patients with AIRDs to understand the characteristics of the immune response to emerging variants such as the Omicron variant, both from a public health perspective and for the development of prime/boost vaccine strategies. Consistent with the result of previous reports,^29^^,^^30^ our study suggests that cellular immunity to SARS-CoV-2 wild-type induced by two doses of BNT162b2 was highly cross-reactive with the Omicron variant in patients with AIRDs as well as in HCs. This cross-reactivity of cellular immunity may be associated with less severe COVID-19 illness during Omicron waves in vaccinated patients with AIRDs.^31^ However, in comparison with HCs, some patients with RA and SLE showed attenuated humoral and cellular immune responses to the Omicron variant. This may be because some immunosuppressant types reduce the vaccine efficacy of either humoral or cellular immunity or both against SARS-CoV-2 variants including Omicron.^9^^,^^26^^,^^32^ Further investigations are needed to demonstrate the impact of different immunosuppressants on humoral and cellular immunity to the Omicron variant.

Our study has some key limitations to consider. Selection bias cannot be excluded because the patients in this registry were selected by a rheumatologist and participated on a voluntary basis. However, as our results, showing that certain immunosuppressants attenuate short-term humoral immune responses, are consistent with those of previous reports,^8^, ^9^, ^10^ we expect that the impact of selection bias on the findings is minimal. The baseline characteristics differ considerably between HCs and patients with AIRDs. However, there are some reports that comorbidities other than age and sex did not affect humoral immunity to BNT162b2.^33^^,^^34^ Therefore, we compared NAb titers adjusted for the effects of age and sex. Additionally, the small sample size is another major limitation of this study. This limitation made it impossible for us to assess the association between vaccine efficacy and disease groups or treatments for all observation periods. Further investigations in a larger cohort are warranted. Finally, we did not evaluate the effects of additional vaccinations after the third dose. Future studies are needed to investigate humoral and cellular immune responses to booster vaccinations in patients with AIRDs.

Overall, our study provides information on the immunogenicity of BNT162b2 in patients with AIRDs and our results suggested that the use of GCs, TNFis, and ABT reduces the duration of the humoral immune response to SARS-CoV-2. In particular, infection with a new VOC, such as the Omicron variant, will likely be particularly harmful to patients with AIRDs because of the insufficient efficacy of two doses of BNT162b2. However, there is insufficient evidence in our data to reach this conclusion. Further studies are needed to reveal the persistent efficacy of the SARS-CoV-2 mRNA vaccine in patients with AIRDs.

## Contributors

Yuta Yamaguchi: Conceptualization, Data curation, Formal analysis, Investigation, Methodology, Resources, Software, Validation, Visualization, Writing – original draft, Writing – review & editing. Shinichiro Nameki: Conceptualization, Data curation, Formal analysis, Investigation, Resources, Validation, Visualization, Writing – original draft, Writing – review & editing. Yasuhiro Kato: Conceptualization, Investigation, Resources, Writing – review & editing. Ryotaro Saita: Formal analysis, Methodology, Software, Writing – original draft, Writing – review & editing. Tomoharu Sato: Formal analysis, Methodology, Software, Writing – review & editing. Sayaka Nagao: Resources. Teruaki Murakami: Resources. Yuko Yoshimine: Resources. Saori Amiya: Resources. Takayoshi Morita: Resources. Yasutaka Okita: Resources. Takahiro Kawasaki: Resources. Jun Fujimoto: Resources. Yasutaka Ueda: Resources. Yuichi Maeda: Resources. Akane Watanabe: Resources. Hyota Takamatsu: Resources. Sumiyuki Nishida: Resources. Yoshihito Shima: Resources. Masashi Narazaki: Resources. Atsushi Kumanogoh: Conceptualization, Funding acquisition, Project administration, Supervision, Writing – review & editing.

## Data sharing statement

Data are available upon reasonable request.

## Declaration of interests

None declared.

## Appendix A Supplementary data

Supplementary Tables S1–S10 and Figs. S1–S3

Caption for supplementary material
