# Supplementary material for: Persistence of SARS-CoV-2 neutralizing antibodies and anti-Omicron IgG induced by BNT162b2 mRNA vaccine in patients with autoimmune inflammatory rheumatic disease: an explanatory study in Japan
Source: Lancet Reg Health West Pac. 2022 Dec 20;32:100661. doi: 10.1016/j.lanwpc.2022.100661 (PMC9763057; doi:10.1016/j.lanwpc.2022.100661)
Supplement: Supplementary Tables S1–S10 and Figs. S1–S3 [file mmc1.docx]

Supplementary appendix:

Persistence of SARS-CoV-2 neutralizing antibodies and anti-Omicron IgG induced by BNT162b2 mRNA vaccine in patients with autoimmune inflammatory rheumatic disease: an explanatory study in Japan

**Table of contents**

[**Supplemental Table 1**. Contingency table of AIRDs and the treatments 2](#_Toc117509254)

[**Supplemental Table 2.** Contingency table of treatments for AIRDs 3](#_Toc117509255)

[**Supplemental Table 3.** Multiple regression analysis of AIRDs 4](#_Toc117509256)

[**Supplemental Table 4.** Contingency table of treatments for rheumatoid arthritis 5](#_Toc117509257)

[**Supplemental Table 5.** Contingency table of treatments for systemic lupus erythematosus 6](#_Toc117509258)

[**Supplemental Table 6.** Contingency table of treatments for ANCA-associated arthritis 7](#_Toc117509259)

[**Supplemental Table 7.** Multiple regression analysis of diagnosis and treatment for rheumatoid arthritis 8](#_Toc117509260)

[**Supplemental Table 8** Multiple regression analysis of treatment for systemic lupus erythematosus 9](#_Toc117509261)

[**Supplemental Table 9.** Demographic and clinical features of patients with ARID and healthy controls measured antigen specific antibody of variants of concern 10](#_Toc117509262)

[**Supplemental Table 10.** Demographic and clinical features of patients with AIRD and healthy controls measured SARS-CoV-2 antigen-specific T-cell responses 12](#_Toc117509263)

[**Supplemental Figure 1.** Serum samples collected from HCs and patients with AIRDs before and after BNT162b2 vaccination. 14](#_Toc117509264)

[**Supplemental Figure 2.** Risk factors for reduced SARS-CoV-2 NAb titers in the treatment of patients with RA, SLE, and AAV. 15](#_Toc117509265)

[**Supplemental Figure 3.** Humoral and cellular responses to SARS-CoV-2 variants in patients with RA and SLE after two doses of BNT162b2. 16](#_Toc117509266)

| **Supplemental Table 1**. Contingency table of AIRDs and the treatments | | | | | | | | | | | |
| --- | --- | --- | --- | --- | --- | --- | --- | --- | --- | --- | --- |
|  | GC | MTX | CyA | TAC | MMF | AZP | TNFis | IL-6Ris | ABT | JAKis | BEL |
| Rheumatoid arthritis | 45 | 117 | 0 | 15 | 0 | 1 | 12 | 33 | 49 | 19 | 0 |
| Systemic lupus erythematosus | 70 | 2 | 7 | 16 | 13 | 6 | 0 | 0 | 0 | 0 | 14 |
| ANCA-associated vasculitis | 31 | 5 | 0 | 0 | 3 | 15 | 0 | 0 | 0 | 0 | 0 |
| Large vessel vasculitis | 12 | 7 | 0 | 1 | 0 | 6 | 0 | 4 | 0 | 0 | 0 |
| Sjögren syndrome | 9 | 2 | 0 | 0 | 1 | 0 | 0 | 0 | 0 | 0 | 0 |
| Systemic sclerosis | 10 | 0 | 0 | 1 | 4 | 1 | 0 | 0 | 0 | 0 | 0 |
| Mixed connective tissue disease | 13 | 2 | 2 | 1 | 3 | 1 | 0 | 0 | 1 | 0 | 0 |
| IgG4 related disease | 9 | 2 | 0 | 0 | 0 | 1 | 0 | 0 | 0 | 0 | 0 |
| Polymyalgia rheumatica | 10 | 2 | 0 | 0 | 0 | 0 | 0 | 1 | 1 | 0 | 0 |
| Behçet disease | 7 | 2 | 0 | 0 | 0 | 0 | 0 | 3 | 0 | 0 | 0 |
| Others^†^ | 14 | 11 | 1 | 2 | 0 | 1 | 1 | 5 | 3 | 0 | 0 |
| ABT, abatacept; ANCA, anti-neutrophil cytoplasmic antibody; AIRD, autoimmune inflammatory rheumatic disease; AZA, azathioprine; BEL, belimumab; CyA, cyclosporine; GC, glucocorticoid; IL-6Ris, interleukin-6 receptor inhibitors; JAKis, Janus kinase inhibitors; MMF, mycophenolate mofetil; MTX, methotrexate; TAC, tacrolimus; TNFis, tumor necrosis factor-α inhibitors. †Adult-onset Still’s disease (n=4), anti-phospholipid antibody syndrome (n=1), ankylosing spondylitis (n=4), common variable immunodeficiency (n=4), polyarteritis nodosa (n=2), psoriatic arthritis (n=5), relapsing polychlorides (n=2), RS3PE syndrome (n=2), SAPHO syndrome (n=2), Castleman disease (n=2), inflammatory myositis (n=6), and necrotizing immune-mediated myopathy (n=1) | | | | | | | | | | | |

| **Supplemental Table 2.** Contingency table of treatments for AIRDs | | | | | | | | | | | |
| --- | --- | --- | --- | --- | --- | --- | --- | --- | --- | --- | --- |
|  | GC | MTX | CyA | TAC | MMF | AZP | TNFis | IL-6Ris | ABT | JAKis | BEL |
| GC | NA | 51 | 10 | 25 | 22 | 29 | 7 | 11 | 15 | 4 | 13 |
| MTX |  | NA | 0 | 6 | 0 | 3 | 7 | 30 | 22 | 11 | 0 |
| CyA |  |  | NA | 0 | 0 | 0 | 0 | 0 | 0 | 0 | 1 |
| TAC |  |  |  | NA | 5 | 0 | 2 | 0 | 6 | 1 | 6 |
| MMF |  |  |  |  | NA | 0 | 0 | 0 | 0 | 0 | 6 |
| AZP |  |  |  |  |  | NA | 0 | 0 | 3 | 0 | 0 |
| TNFis |  |  |  |  |  |  | NA | 0 | 0 | 0 | 0 |
| IL-6Ris |  |  |  |  |  |  |  | NA | 0 | 0 | 0 |
| ABT |  |  |  |  |  |  |  |  | NA | 0 | 0 |
| JAKis |  |  |  |  |  |  |  |  |  | NA | 0 |
| BEL |  |  |  |  |  |  |  |  |  |  | NA |
| ABT, abatacept; AIRD, autoimmune inflammatory rheumatic disease; AZA, azathioprine; BEL, belimumab; CyA, cyclosporine; GC, glucocorticoid; IL-6Ris, interleukin-6 receptor inhibitors; JAKis, Janus kinase inhibitors; MMF, mycophenolate mofetil; MTX, methotrexate; TAC, tacrolimus; TNFis, tumor necrosis factor-α inhibitors; NA, not applicable. | | | | | | | | | | | |

| Supplemental Table 3. Multiple regression analysis of AIRDs | | | | | | |  |
| --- | --- | --- | --- | --- | --- | --- | --- |
|  | **Term 1 (n=527)** | | **Term 2 (n=396)** | | **Term 3 (n=402)** | |  |
|  | Regression coefficient | p value | Regression coefficient | p value | Regression coefficient | p value |  |
| Intercept | 0.5464 | <0·0001 | 3.237 | <0·0001 | 2.168 | <0·0001 |  |
| Age | 0·0000368 | 0·9644 | –0·0117 | <0·0001 | –0·0100 | <0·0001 |  |
| Sex (male) | 0·0087 | 0·6948 | –0·1002 | 0·1462 | –0·0959 | 0·1764 |  |
| Rheumatoid arthritis | 0·0857 | 0·0112 | –0·5535 | <0·0001 | –0·2550 | 0·1031 |  |
| Systemic lupus erythematosus | 0·0365 | 0·2899 | –0·5099 | <0·0001 | –0·2596 | 0·0914 |  |
| ANCA-associated vasculitis | 0·1089 | 0·0303 | –0·9051 | <0·0001 | –0·4422 | 0·0145 |  |
| Large vessel vasculitis | 0·026 | 0·6981 | –0·4694 | 0·0472 | –0·3144 | 0·1269 |  |
| Sjögren syndrome | 0·0759 | 0·2458 | 0·1318 | 0·5047 | 0·001 | 0·9960 |  |
| Systemic sclerosis | 0·1185 | 0·0553 | –0·4059 | 0·0230 | –0·0691 | 0·7342 |  |
| Mixed connective tissue disease | –0·0077 | 0·9064 | –0·0606 | 0·8243 | –0·0029 | 0·9889 |  |
| IgG4 related disease | 0·0092 | 0·9098 | 0·0971 | 0·7040 | –0·0332 | 0·8821 |  |
| Polymyalgia rheumatica | 0·1238 | 0·1252 | 0·0367 | 0·8910 | –0·0782 | 0·7443 |  |
| Behçet disease | 0·0633 | 0·3791 | –0·3314 | 0·1814 | –0·0549 | 0·8065 |  |
| Others^†^ | 0·0248 | 0·5846 | –0·6440 | <0·0001 | –0·1796 | 0·2977 |  |
| ANCA, anti-neutrophil cytoplasmic antibody; Term 1, pre-vaccination; Term 2, 14–42 days after second vaccination; Term 3, 100–200 days after second vaccination. †Adult-onset Still’s disease (n=4), anti-phospholipid antibody syndrome (n=1), ankylosing spondylitis (n=4), common variable immunodeficiency (n=4), polyarteritis nodosa (n=2), psoriatic arthritis (n=5), relapsing polychlorides (n=2), RS3PE syndrome (n=2), SAPHO syndrome (n=2), Castleman disease (n=2), inflammatory myositis (n=6), and necrotizing immune-mediated myopathy (n=1) | | | | | | |  |
|  |  |  |  |  |  |  |  |
|  |  |  |  |  |  |  |  |

| Supplemental Table 4. Contingency table of treatments for rheumatoid arthritis | | | | | | | | |  |
| --- | --- | --- | --- | --- | --- | --- | --- | --- | --- |
|  | GC | MTX | TAC | AZP | TNFis | IL-6Ris | ABT | JAKis |  |
| GC | NA | 26 | 5 | 1 | 10 | 8 | 4 | 7 |  |
| MTX |  | NA | 4 | 1 | 27 | 19 | 11 | 6 |  |
| TAC |  |  | NA | 0 | 0 | 6 | 1 | 2 |  |
| AZA |  |  |  | NA | 0 | 0 | 0 | 0 |  |
| TNFis |  |  |  |  | NA | 0 | 0 | 0 |  |
| IL-6Ris |  |  |  |  |  | NA | 0 | 0 |  |
| ABT |  |  |  |  |  |  | NA | 0 |  |
| JAKis |  |  |  |  |  |  |  | NA |  |
| ABT, abatacept; AZA, azathioprine; GC, glucocorticoid; IL-6Ris, interleukin-6 receptor inhibitors; JAKis, Janus kinase inhibitors; MTX, methotrexate; TAC, tacrolimus; TNFis, tumor necrosis factor-α inhibitors; NA, not applicable. | | | | | | | | |  |
|  |  |  |  |  |  |  |  |  |  |
|  |  |  |  |  |  |  |  |  |  |
|  |  |  |  |  |  |  |  |  |  |

| Supplemental Table 5. Contingency table of treatments for systemic lupus erythematosus | | | | | | | |  |
| --- | --- | --- | --- | --- | --- | --- | --- | --- |
|  | GC | MTX | MMF | TAC | CyA | AZP | BEL |  |
| GC | NA | 2 | 12 | 15 | 7 | 5 | 13 |  |
| MTX |  | NA | 0 | 0 | 0 | 0 | 0 |  |
| MMF |  |  | NA | 4 | 0 | 0 | 6 |  |
| TAC |  |  |  | NA | 0 | 0 | 6 |  |
| CyA |  |  |  |  | NA | 0 | 1 |  |
| AZA |  |  |  |  |  | NA | 0 |  |
| BEL |  |  |  |  |  |  | NA |  |
| AZA, azathioprine; BEL, belimumab; CyA, cyclosporine; GC, glucocorticoid; MMF, mycophenolate mofetil; MTX, methotrexate; TAC, tacrolimus; NA, not applicable. | | | | | | | |  |
|  |  |  |  |  |  |  |  |  |
|  |  |  |  |  |  |  |  |  |

| Supplemental Table 6. Contingency table of treatments for ANCA-associated arthritis | | | | |  |
| --- | --- | --- | --- | --- | --- |
|  | GC | MTX | AZP | MMF |  |
| GC | NA | 5 | 15 | 3 |  |
| MTX |  | NA | 0 | 0 |  |
| AZA |  |  | NA | 0 |  |
| MMF |  |  |  | NA |  |
| ANCA, anti-neutrophil cytoplasmic antibody; AZA, azathioprine; GC, glucocorticoid; MMF, mycophenolate mofetil; MTX, methotrexate; NA, not applicable. | | | | |  |
|  |  |  |  |  |  |
|  |  |  |  |  |  |

| Supplemental Table 7. Multiple regression analysis of diagnosis and treatment for rheumatoid arthritis | | | | | | |  |
| --- | --- | --- | --- | --- | --- | --- | --- |
|  | **Term 1 (n=317)** | | **Term 2 (n=253)** | | **Term 3 (n=190)** | |  |
|  | Regression coefficient | p value | Regression coefficient | p value | Regression coefficient | p value |  |
| Intercept | 0.4090 | <0·0001 | 3.3411 | <0·0001 | 2.1854 | <0·0001 |  |
| Rheumatoid arthritis | -0.0521 | 0.3354 | -0.1208 | 0.3515 | 0.0470 | 0.7873 |  |
| Age | 0.0029 | 0.0103 | -0.0150 | <0·0001 | -0.0108 | 0.0004 |  |
| Male | 0.0456 | 0.0943 | -0.0520 | 0.4014 | -0.0761 | 0.3856 |  |
| Glucocorticoids | 0.0149 | 0.7060 | -0.4365 | <0·0001 | -0.2011 | 0.0222 |  |
| Methotrexate | 0.0411 | 0.2649 | -0.1800 | 0.0602 | -0.1324 | 0.1111 |  |
| Tacrolimus | -0.0222 | 0.7134 | 0.1046 | 0.5399 | 0.1175 | 0.4000 |  |
| TNF-α inhibitors | 0.0660 | 0.1617 | -0.0506 | 0.6980 | -0.3417 | 0.0019 |  |
| IL-6R inhibitors | 0.0815 | 0.0615 | -0.0523 | 0.6470 | -0.0579 | 0.5537 |  |
| Abatacept | 0.0756 | 0.2135 | -0.6531 | <0·0001 | -0.5408 | <0·0001 |  |
| JAK inhibitors | 0.0989 | 0.1536 | -0.1910 | 0.3128 | -0.2850 | 0.0647 |  |
| IL-6R, interleukin-6 receptor; JAK, Janus kinase; TNF, tumor necrosis factor; Term 1, pre-vaccination; Term 2, 14–42 days after second vaccination; Term 3, 100–200 days after second vaccination. | | | | | | |  |
|  |  |  |  |  |  |  |  |

| **Supplemental Table 8** Multiple regression analysis of treatment for systemic lupus erythematosus | | | | | | |
| --- | --- | --- | --- | --- | --- | --- |
|  | **Term 1 (n=208)** | | **Term 2 (n=193)** | | **Term 3 (n=81)** | |
|  | Regression coefficient | p value | Regression coefficient | p value | Regression coefficient | p value |
| Intercept | 0.5604 | <0·0001 | 3.1187 | <0·0001 | 2.2713 | <0·0001 |
| Age | -0.0006 | 0.7031 | -0.0101 | 0.0014 | -0.0176 | 0.0006 |
| Male | 0.0307 | 0.3860 | -0.0054 | 0.9343 | 0.2754 | 0.1054 |
| Glucocorticoids | 0.0604 | 0.2303 | -0.3248 | 0.0019 | 0.1064 | 0.5579 |
| Tacrolimus | -0.0077 | 0.9176 | 0.0468 | 0.7511 | -0.0882 | 0.6249 |
| Cyclosporine | 0.0596 | 0.5613 | 0.0639 | 0.7584 | -0.2113 | 0.3237 |
| Mycophenolate mofetil | 0.1068 | 0.1972 | -0.6704 | 0.0003 | -0.4434 | 0.0234 |
| Belimumab | -0.1537 | 0.0722 | -0.4403 | 0.0078 | -0.2981 | 0.1340 |
| Term 1, pre-vaccination; Term 2, 14–42 days after second vaccination; Term 3, 100–200 days after second vaccination. | | | | | | |

| Supplemental Table 9. Demographic and clinical features of patients with ARID and healthy controls measured antigen specific antibody of variants of concern | | | |
| --- | --- | --- | --- |
|  | **RA (n=66)** | **SLE (n=26)** | **HC (n=13)** |
| Demographic characteristics |  |  |  |
| Age (years), median (IQR [range]) | 72 (62 – 78 [40–85]) | 50 (40·8–61·5 [27–71]) | 35 (31·5–43·5 [27–51]) |
| Male | 12 (18·2) | 4 (15·4) | 6 (46·2) |
| BMI (kg/m^2^), median (IQR) | 21·2 (19·1–24) | 22·5 (19·9–25·9) | 22·2 (18·5–25·2) |
| Treatment for AIRDs |  |  |  |
| Glucocorticoid | 22 (33·3) | 25 (96·2) | – |
| dose (mg/day), median (IQR) | 4 (1·75–5·25) | 5 (4–6·75) | – |
| Methotrexate | 36 (54·5) | 1 (3·9) | – |
| dose (mg/week), median (IQR) | 6 (4–8) | 8 (8–8) | – |
| Cyclosporine | 0 (0) | 3 (11·5) | – |
| dose (mg/day), median (IQR) | NA | 25 (10–120) | – |
| Tacrolimus | 6 (9·1) | 8 (30·8) | – |
| dose (mg/day), median (IQR) | 1 (1–2) | 2·5 (2–2·88) | – |
| Mycophenolate mofetil | 0 (0) | 5 (19·2) | – |
| dose (mg/day), median (IQR) | NA | 1500 (1000–1750) | – |
| Azathioprine | 1 (1·5) | 4 (15·4) | – |
| dose (mg/day), median (IQR) | 50 (50–50) | 50 (50–87·5) | – |
| TNF-α inhibitors | 11 (16·7) | 0 (0) | – |
| IL-6R inhibitors | 19 (28·8) | 0 (0) | – |
| Abatacept | 10 (15·2) | 0 (0) | – |
| JAK inhibitors | 4 (6·1) | 0 (0) | – |
| Belimumab | 0 (0) | 4 (15·4) | – |
| No therapy | 4 (6·1) | 0 (0) | – |
| n (%) presented unless otherwise specified. | | | |
| AIRD, autoimmune inflammatory rheumatic disease; BMI, body mass index; HC, healthy control; IL-6R, interleukin-6 receptor; IQR, interquartile range; JAK, Janus kinase; RA, rheumatoid arthritis; SLE, systemic lupus erythematosus; TNF, tumor necrosis factor; Term1, pre-vaccination; Term2, 14–42 days after second vaccination; Term3, 100–200 days after second vaccination; NA, not applicable. | | | |

| Supplemental Table 10. Demographic and clinical features of patients with AIRD and healthy controls measured SARS-CoV-2 antigen-specific T-cell responses | | | |
| --- | --- | --- | --- |
|  | **RA (n=37)** | **SLE (n=11)** | **HC (n=15)** |
| Demographic characteristics |  |  |  |
| Age (years), median (IQR [range]) | 68 (53·5–76·5 [32–83]) | 49 (39–65 [32–68]) | 34 (31–37 [30–49]) |
| Male | 4 (10·8) | 1 (9·1) | 10 (66·7) |
| BMI (kg/m^2^), median (IQR) | 21·1 (19·1–23·1) | 23·9 (13·2–26·7) | 23·0 (20·1–24·6) |
| Treatment for AIRDs |  |  |  |
| Glucocorticoid | 6 (16·2) | 10 (90·9) | – |
| dose (mg/day), median (IQR) | 3·75 (1·75–9·5) | 4·5 (2·38–6·25) | – |
| Methotrexate | 24 (64·9) | 1 (9·1) | – |
| dose (mg/week), median (IQR) | 7 (6–9·5) | 8 (8–8) | – |
| Cyclosporine | 0 (0) | 0 (0) | – |
| dose (mg/day), median (IQR) | NA | NA | – |
| Tacrolimus | 2 (5·4) | 3 (27·3) | – |
| dose (mg/day), median (IQR) | 1·25 (1–1·5) | 2·5 (2–3) | – |
| Mycophenolate mofetil | 0 (0) | 2 (18·2) | – |
| dose (mg/day), median (IQR) | NA | 1000 (500–1500) | – |
| Azathioprine | 0 (0) | 1 (9·1) | – |
| dose (mg/day), median (IQR) | NA | 50 (50–50) | – |
| TNF-α inhibitors | 7 (18·9) | 0 (0) | – |
| IL-6R inhibitors | 8 (21·6) | 0 (0) | – |
| Abatacept | 6 (16·2) | 0 (0) | – |
| JAK inhibitors | 2 (5·4) | 0 (0) | – |
| Belimumab | 0 (0) | 1 (9·1) | – |
| No therapy | 0 (0) | 0 (0) | – |
| n (%) presented unless otherwise specified. | | | |
| AIRD, autoimmune inflammatory rheumatic disease; BMI, body mass index; HC, healthy control; IL-6R, interleukin-6 receptor; IQR, interquartile range; JAK, Janus kinase; RA, rheumatoid arthritis; SLE, systemic lupus erythematosus; TNF, tumor necrosis factor; NA, not applicable. | | | |

**
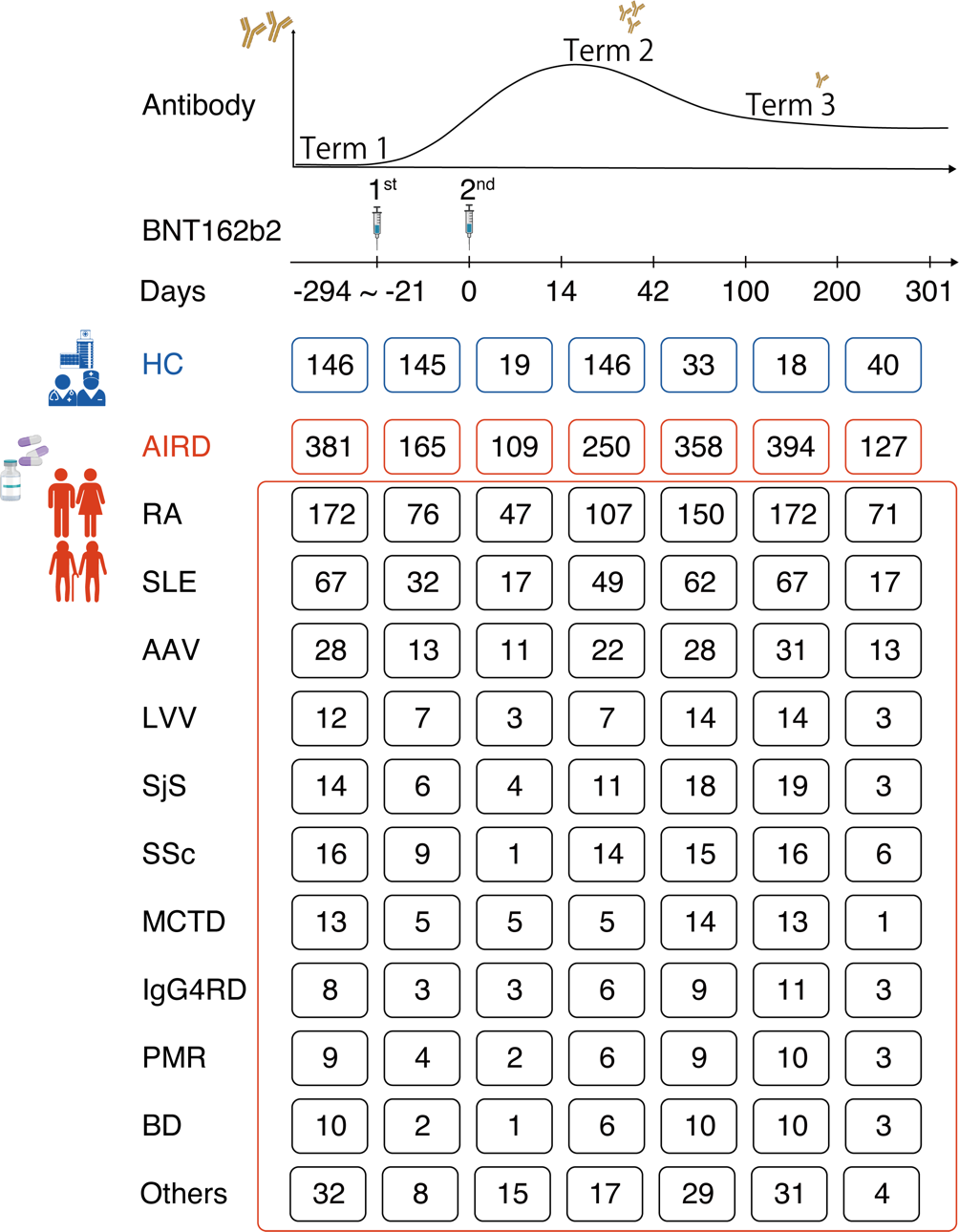
**

# Supplemental Figure 1. Serum samples collected from HCs and patients with AIRDs before and after BNT162b2 vaccination. HCs and patients with AIRDs are marked by blue and red, respectively. The X-axis represents the day after the second vaccination with BNT162b2. Term 1, before first vaccination; Term 2, 14–42 days after second vaccination; Term 3, 100–200 days after second vaccination. AAV, anti-neutrophil cytoplasmic antibody-associated vasculitis; AIRD, autoimmune inflammatory rheumatic disease; BD, Behçet disease; HC, healthy control; IgG4RD, IgG4-related disease; LVV, large vessel vasculitis; MCTD, mixed connective tissue disease; PMR, polymyalgia rheumatica; RA, rheumatoid arthritis; SjS, Sjögren syndrome; SLE, systemic lupus erythematosus; SSc, systemic sclerosis. The diagnoses of adult-onset Still’s disease, anti-phospholipid antibody syndrome, ankylosing spondylitis, common variable immunodeficiency, polyarteritis nodosa, psoriatic arthritis, relapsing polychondritis, RS3PE syndrome, SAPHO syndrome, Castleman disease, and necrotizing immune-mediated myopathy were included in the others group.


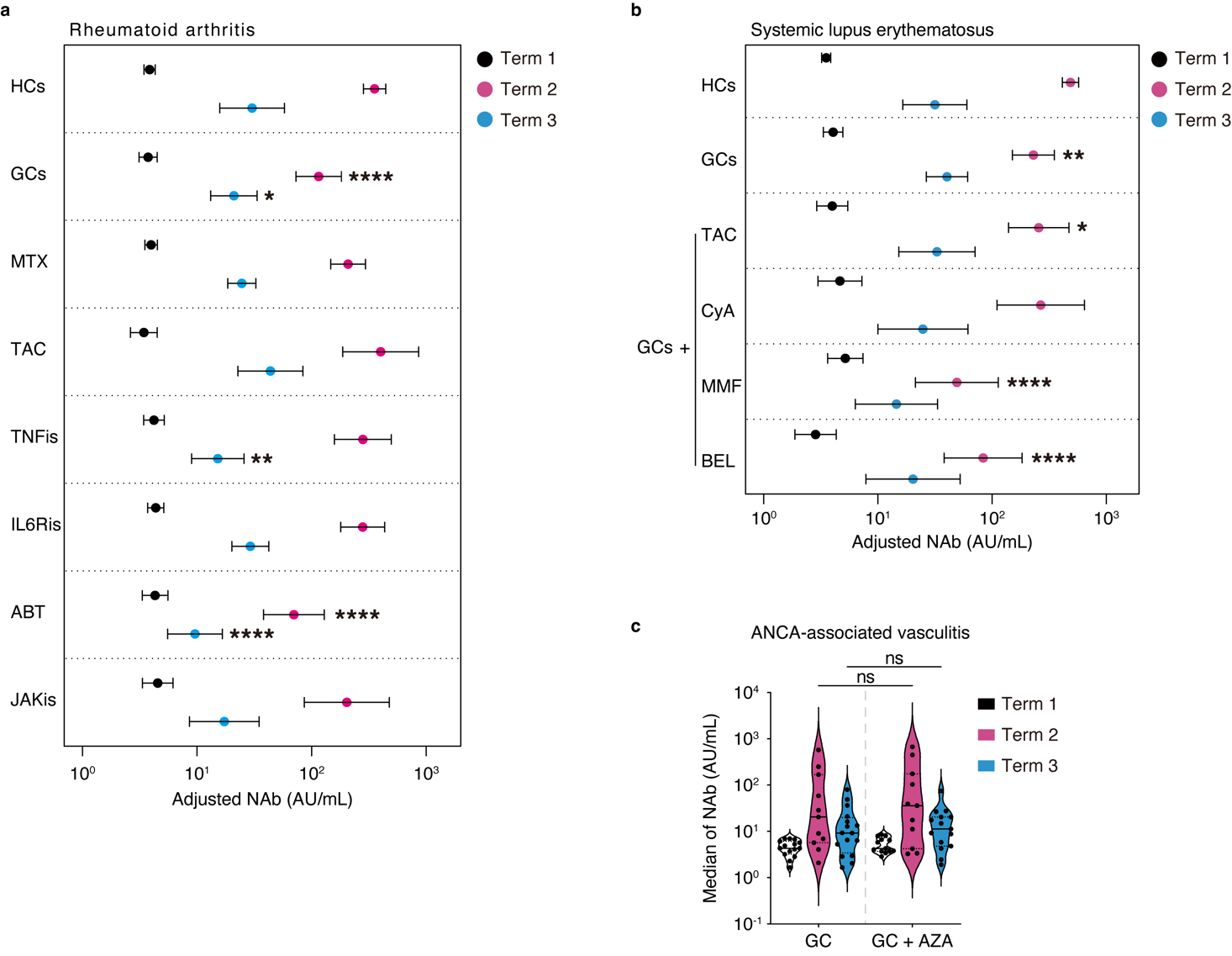


# Supplemental Figure 2. Risk factors for reduced SARS-CoV-2 NAb titers in the treatment of patients with RA, SLE, and AAV. (a, b) Adjusted mean values of SARS-CoV-2 NAb (adjusted NAb) titers induced by BNT162b2 in patients with RA (Term 1, n=172; Term 2, n=107; Term 3, n=172) taking into account age, sex, diagnosis of RA, and treatment covariate (a) or SLE (Term 1, n=67; Term 2, n=49; Term 3, n=67) taking into account age, sex, and treatment covariates (b). The means were adjusted following multiple regression analysis with the reference of NAb titers in healthy controls. The X-axis is represented on a log scale. The error bar indicates the 95% CI. An unpaired Student's t test was performed to compare each covariate with HCs. *p<0·05, **p<0·01, ****p<0·0001. (c) The comparison of NAb titers induced by BNT162b2 in patients undergoing GC monotherapy (Term1, n=8; Term2, n=6; Term3, n=8) and those undergoing treatment with GCs and AZA (Term1, n=13; Term2, n=11; Term3, n=15). The X-axis is represented on a log scale. Error bars indicate interquartile ranges. Mann-Whitney U test was used to evaluate the significant differences between the groups. ns, not significant. ABT, abatacept; ANCA, anti-neutrophil cytoplasmic antibody; AZA, azathioprine; BEL, belimumab; CTRL, controls; CyA, cyclosporine A; GC, glucocorticoid; HC, healthy control; IL-6Ris, interleukin (IL)-6 receptor inhibitors; JAKis, Janus kinase inhibitors; MMF, mycophenolate mofetil; MTX, methotrexate; RA, rheumatoid arthritis; SLE, systemic lupus erythematosus; TAC, tacrolimus; TNFis, tumor necrosis factor-α inhibitors. Term 1 (black), pre-vaccination; Term 2 (red), 14–42 days after the second vaccination; Term 3 (blue), 100–200 days after the second vaccination.

**
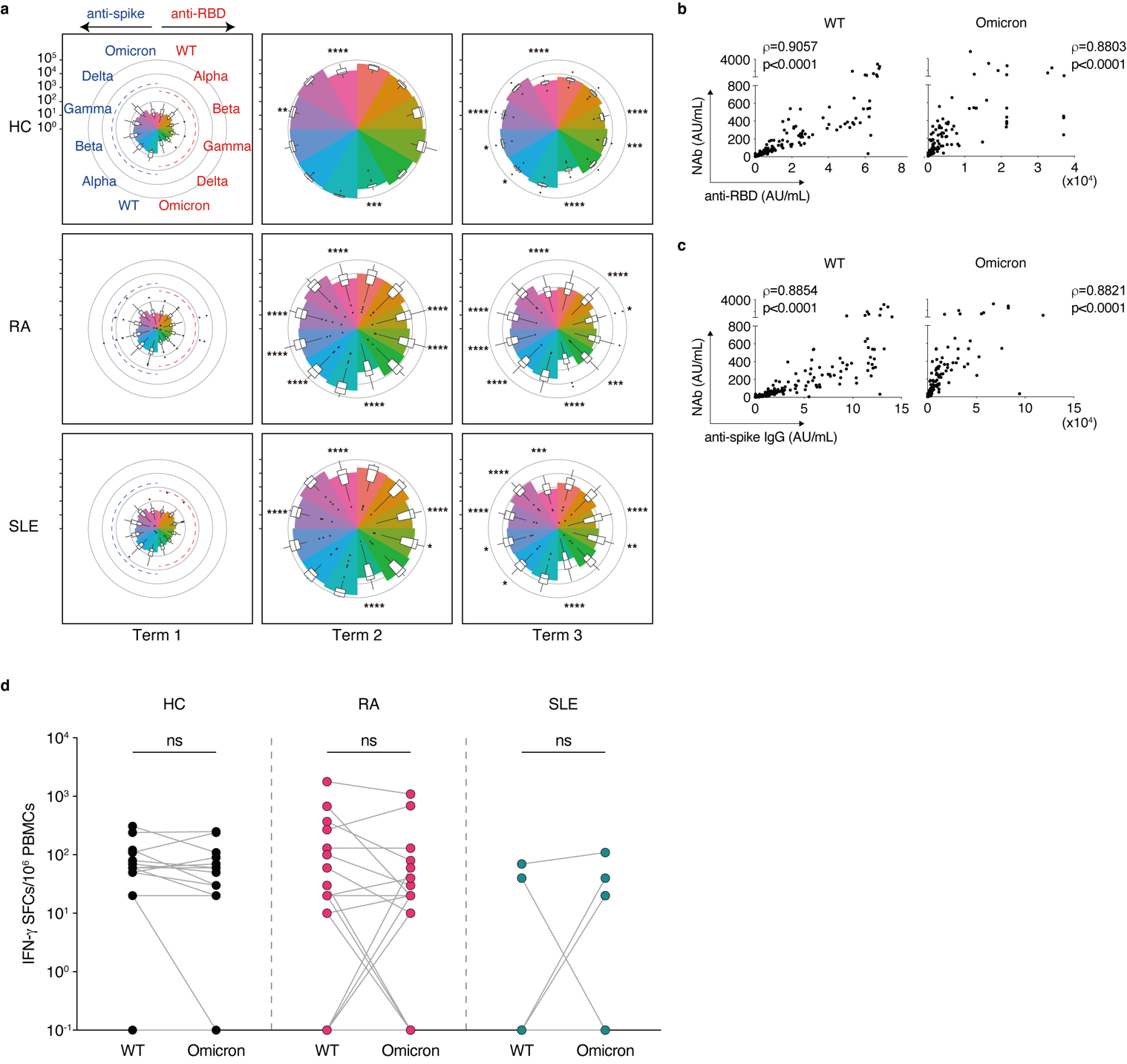
**

# Supplemental Figure 3. Humoral and cellular responses to SARS-CoV-2 variants in patients with RA and SLE after two doses of BNT162b2. (a) Nightingale rose diagram of median antigen-specific IgG levels against SARS-CoV-2 RBD or Spike (WT and VOCs for each antigen) in HCs (n=13, upper), patients with RA (n=66, middle), and patients with SLE (n=26, lower). The boxplots show the medians (middle line) and the first and third quartiles, while the whiskers indicate 95% interquartile ranges. Each circle in the diagram represents logarithmically increasing concentrations from 100–105 AU/mL, as shown for the HCs (upper left). Dotted semicircles marked by red or blue indicate the cut-off value for anti-WT RBD IgG (538 AU/mL) and anti-WT Spike IgG (1,960 AU/mL) positivity, respectively, according to the manufacturer’s protocol. Term 1 (left), pre-vaccination; Term 2 (center), 14–42 days after the second vaccination; Term 3 (right), 100–200 days after the second vaccination. Friedman and Dunn’s multiple comparisons tests were performed to evaluate the significance of differences of anti-VOC RBD and Spike IgG titers compared to anti-WT RBD and Spike IgG titers, respectively. *p<0·05, **p<0·01, ***p<0·001, ****p<0·0001. (b, c) Correlation of the serum concentrations of anti-SARS-CoV-2 RBD (b) and Spike protein (c) IgG titers with the SARS-CoV-2 NAb titers (n=105, for each Term). Left, anti-WT IgG; right, anti-Omicron IgG. Spearman’s test was used to evaluate the significance of differences. (d) Medium-Term T-cell responses to SARS-CoV-2 WT and Omicron spike protein peptides in HCs (n=15, left, black) and patients with RA (n=32, center, red) or SLE (n=10, right, blue) at Term 3. The Y-axis represents a log scale. For the visualization of data on the log scale, values=0 are represented by 0·1. The Wilcoxon matched-pairs signed rank test was performed to evaluate the significant differences in T-cell responses to Omicron spike protein peptides comparing with WT. ns, not significant. HC, healthy control; PBMC, peripheral blood mononuclear cell; RA, rheumatoid arthritis; RBD, receptor-binding domain; SLE, systemic lupus erythematosus; Spike, SARS-CoV-2 Spike protein; SFC, spot-forming cell; VOC, variant of concern (Alpha, B.1.17; Beta, B.1.351; Gamma, P.1; Delta, B.1.617.2; Omicron, B.1.1529); WT, SARS-CoV-2 wild type.
