# Supplementary material for: Persistence of SARS-CoV-2 neutralizing antibodies and anti-Omicron IgG induced by BNT162b2 mRNA vaccine in patients with autoimmune inflammatory rheumatic disease: an explanatory study in Japan
Source: Lancet Reg Health West Pac. 2022 Dec 20;32:100661. doi: 10.1016/j.lanwpc.2022.100661 (PMC9763057; doi:10.1016/j.lanwpc.2022.100661)
Supplement: Caption for supplementary material [file mmc2.docx]

Supplementary appendix:

Persistence of SARS-CoV-2 neutralizing antibodies and anti-Omicron IgG induced by BNT162b2 mRNA vaccine in patients with autoimmune inflammatory rheumatic disease: an explanatory study in Japan

**Table of contents**

**Supplemental Table 1.** Contingency table of AIRDs and the treatments. 2

**Supplemental Table 2.** Contingency table of treatments for AIRDs. 3

**Supplemental Table 3.** Multiple regression analysis of AIRDs. 4

**Supplemental Table 4.** Contingency table of treatments for rheumatoid arthritis. 5

**Supplemental Table 5.** Contingency table of treatments for systemic lupus erythematosus. 6

**Supplemental Table 6.** Contingency table of treatments for ANCA-associated arthritis. 7

**Supplemental Table 7.** Multiple regression analysis of diagnosis and treatment for rheumatoid arthritis. 8

**Supplemental Table 8** Multiple regression analysis of treatment for systemic lupus erythematosus. 9

**Supplemental Table 9.** Demographic and clinical features of patients with ARID and healthy controls measured antigen specific antibody of variants of concern. 10

**Supplemental Table 10.** Demographic and clinical features of patients with AIRD and healthy controls measured SARS-CoV-2 antigen-specific T-cell responses. 12

**Supplemental Figure 1.** Serum samples collected from HCs and patients with AIRDs before and after BNT162b2 vaccination. 14

**Supplemental Figure 2.** Risk factors for reduced SARS-CoV-2 NAb titers in the treatment of patients with RA, SLE, and AAV. 15

**Supplemental Figure 3.** Humoral and cellular responses to SARS-CoV-2 variants in patients with RA and SLE after two doses of BNT162b2. 16
